# Clustered Regularly Interspaced Short Palindromic Repeats (CRISPR)-Based Genome Editing in Inherited Cardiovascular Diseases: Therapeutic Advances, Emerging Editing Platforms, and Translational Challenges

**DOI:** 10.7759/cureus.116111

**Published:** 2026-09-11

**Authors:** Tosin Fakiyesi, Mmaduabuchi Ozioko, Oluwapelumi D Ogunseye, Olaoluwa Adeyemi, Ibukunoluwa Adedoyin, Adeolu A Badejo, Olorungbami Anifalaje, Abiodun Adegbesan, Oluwakemi Olalude, Rachael Tiamiyu, Almustapha Hassan

**Affiliations:** 1 General Medicine, Shirley Oaks Hospital, Croydon, GBR; 2 Cardiology, Leeds Teaching Hospitals NHS Trust, Leeds, GBR; 3 Public Health, University of Ibadan, Ibadan, NGA; 4 Internal Medicine, Advocate Christ Medical Center, Oak Lawn, USA; 5 Internal Medicine, University of Ilorin Teaching Hospital, Ilorin, NGA; 6 Trauma and Orthopaedic Surgery, Newcastle Upon Tyne Hospitals NHS Foundation Trust, Newcastle, GBR; 7 Plastic and Reconstructive Surgery, Mid Yorkshire Hospitals NHS Trust, Wakefield, GBR; 8 Menzies Institute for Medical Research, University of Tasmania, Tasmania, AUS; 9 Internal Medicine, Lagos State University Teaching Hospital, Lagos, NGA; 10 Biochemistry, Wayne State University, Detroit, USA; 11 Internal Medicine, Usman Danfodiyo University Teaching Hospital, Sokoto, NGA

**Keywords:** base editing, cardiac channelopathies, dilated cardiomyopthy, epigenome editing, familial hypercholestrolemia, genome editing, hypertrophic cardiomyopathy (hcm), inherited cardiovascular diseases, prime editing, crispr-cas9

## Abstract

Inherited cardiovascular diseases (ICVDs) are a broad category of hereditary disorders resulting from pathogenic mutations that alter cardiac anatomy, electrical conduction, and metabolic pathways. The main types include hypertrophic cardiomyopathy (HCM), dilated cardiomyopathy (DCM), cardiac channelopathies, and familial hypercholesterolemia (FH) and are associated with heart failure, malignant arrhythmias, and sudden cardiac death, particularly in young people. Current treatments, such as pharmacological therapies, implantable cardioverter-defibrillators, and heart transplantation, have significantly improved outcomes; however, these treatments are mainly targeted toward symptom control and the prevention of complications without correcting the underlying genetic defects. Recent advancements in genome editing have transformed precision cardiovascular care, with clustered regularly interspaced short palindromic repeats (CRISPR)-based technologies emerging as potential methods for targeting and modifying disease-causing variants. Emerging platforms, such as base editing, prime editing, and epigenome editing, have widened the therapeutic potential of genome engineering by enhancing editing precision and reducing some of the constraints of the traditional CRISPR-Cas9 strategy. In the setting of inherited cardiovascular disorders, promising early results have been obtained in preclinical studies using human induced pluripotent stem cell and animal models, including correction of *MYBPC3* and *MYH7* mutations in HCM; targeting of *TTN*-, *RBM2*0-, and *LMNA*-associated mechanisms in DCM; restoration of ion channel function in inherited arrhythmia syndromes; and modulation of lipid-related genes such as *PCSK9*, *LDLR*, and *ANGPTL3* in FH. However, major challenges remain, including off-target effects, effective delivery to cardiac tissues, immunological responses, interpretation of genetic variants, and ethical concerns. This review provides a translational, integrated view of the evolution of CRISPR-based genome editing technologies, their application to ICVDs, current preclinical and clinical advances, and the challenges that need to be addressed to achieve safe and effective translation into cardiovascular therapeutics.

## Introduction and background

Inherited cardiovascular diseases (ICVDs) are a group of disorders caused by mutations in genes that are important for cardiac structure and function. Cardiovascular diseases constitute a major health burden globally. The primary ICVDs include hypertrophic cardiomyopathy (HCM), dilated cardiomyopathy (DCM), cardiac channelopathies, including long QT syndrome and Brugada syndrome, and familial hypercholesterolemia (FH) [1]. These conditions may lead to heart failure and sudden cardiac death, particularly in young individuals and those with pathogenic genetic mutations. Although there has been considerable progress in understanding the genetic basis of these disorders, ICVDs remain prevalent, with estimates suggesting that roughly 3% of the general population may be affected [2]. Current therapies, such as beta blockers, ACE inhibitors, and statins, have been useful in clinical management but do not repair pathogenic mutations [2]. In advanced cases, implantable cardioverter defibrillators (ICDs) are beneficial in preventing lethal arrhythmias, but they do not alter the underlying pathophysiology of the condition. Although heart transplantation may be curative, it can be restricted by the availability of organs and the need for lifelong immunosuppressants [3]. Due to the limits of these traditional techniques, there is a need for innovative treatment strategies that can target and rectify genetic mutations [3]. This progress is being driven by clustered regularly interspaced short palindromic repeats (CRISPR)-Cas9, as genome editing has quickly matured both as a research tool and as a therapeutic option [4]. CRISPR-Cas9 is based on a bacterial adaptive immune mechanism and uses a guide RNA to target Cas9 to a specific genomic location to create a double-strand break. The cell’s own repair machinery either disrupts the gene or, in the presence of a suitable template, installs a specific sequence change. It is easier to design and more readily programmable than earlier programmable nucleases, such as zinc-finger nucleases and transcription activator-like effector nucleases (TALENs) [5].

Interestingly, the field of genome editing has expanded beyond double-strand cleavage. Base editors are composed of a DNA-targeting module and a deaminase, engineered to provide precise nucleotide conversion without generating double-strand breaks. Prime editors expand on this by fusing a Cas-derived nickase with a reverse transcriptase and using a prime-editing guide RNA (pegRNA) to directly write new sequences at the target site, in principle enabling a wider range of substitutions and small indels than base editing alone, although real-world efficiency still heavily depends on target context, guide design, and delivery [6]. Additional strategies to modulate expression, alter epigenetic marks, or insert DNA at defined sites without conventional cleavage include CRISPR activation/interference, epigenome editing, RNA editing, and CRISPR-associated transposases [7, 8]. However, these advances have not eliminated the practical barriers. Conventional CRISPR-Cas9 induces double-strand breaks that can cause off-target mutations, unintended indels, and larger structural rearrangements [9]. The efficiency of editing is also limited by the availability of suitable protospacer-adjacent motifs, immune responses to editing components or delivery vectors, and, perhaps most fundamentally for cardiovascular applications, the difficulty of safely and selectively delivering these tools to cardiac tissue [10]. The challenge for ICVD in particular is to durably correct pathogenic variants with minimal unintended genomic alterations in cardiomyocytes and other relevant tissues. Despite these challenges, the clinical picture is moving faster than we realize; CRISPR and similar genome-editing therapies have already entered early human trials for some ICVDs, such as FH and transthyretin amyloid cardiomyopathy [11, 12]. However, this clinical progress should not be interpreted as evidence that genome editing has emerged as an established clinical tool across all ICVDs. For many monogenic cardiomyopathies and channelopathies, including HCM, DCM, long QT syndrome, and Brugada syndrome, it remains predominantly at the preclinical stage [12], and questions regarding long-term safety, durability, tissue-specific delivery, off-target risk, immunogenicity, and the broader consequences of permanent genomic modification remain largely unanswered [12].

Prior reviews have generally focused either on the general principles of genome editing or on a single cardiovascular disease in isolation. Here, we take a more integrated approach, connecting the genetic architecture of ICVD with the trajectory of CRISPR-based disease modelling and therapy. We discuss the development of editing platforms, from traditional CRISPR-Cas9 to base editing, prime editing, and epigenome editing, and their application in cardiomyopathies, channelopathies, and FH. We critically appraise emerging strategies for cardiovascular-targeted delivery and the preclinical and early clinical evidence, with a focus on the biological, technical, ethical, and translational barriers between current progress and broader clinical use.

Methods

A systematic literature search was performed in PubMed/MEDLINE, Scopus, Web of Science, Embase, and Google Scholar for papers published between 2002 and 2026. We searched using a combination of Medical Subject Headings (MeSH) and free-text terms linked with Boolean operators (“AND” and “OR”). Original experimental studies, preclinical animal studies, human induced pluripotent stem cell (iPSC) studies, early-phase clinical trials, translational research, systematic reviews, narrative reviews, consensus statements, and clinical practice guidelines published in English were eligible. Special emphasis was placed on high-quality research evaluating CRISPR-mediated therapeutic correction of inherited cardiomyopathies, cardiac channelopathies, and FH. The exclusion criteria were non-cardiovascular disorders, genome-editing methods unrelated to the topic, conference abstracts without full-text publications, editorials or commentaries, and non-English papers unless they contained groundbreaking data unavailable elsewhere. The retrieved papers were screened for scientific relevance, methodological quality, novelty, and translational importance. Evidence was synthesised thematically rather than quantitatively.

## Review

Overview of CRISPR-Cas9 gene editing

The term “CRISPR” was coined in 2002, although the narrative starts in 1987, when unusual palindromic repeat sequences with non-repeating spacers were found in *Escherichia coli *[13]. Similar patterns were subsequently identified in numerous archaeal and bacterial species. However, the biological relevance of these sequences was not clear until 2005, when three separate studies proved that the spacer sequences were obtained from foreign bacteriophage DNA. This finding was crucial because it helped establish their connection to adaptive immunity [14]. The CRISPR-associated (Cas) genes were first reported in 2002. Some of these genes were shown to encode proteins containing helicase and nuclease motifs. CRISPR-Cas systems are classified into two classes, Class 1 and Class 2. Class 1 systems usually work via multiprotein effector complexes, while Class 2 systems depend on a single multidomain effector protein. Cas9 is a Class 2 (Type II) member that requires just a single effector protein and is directed by two RNAs, crRNA and tracrRNA. Together, these components enable the system to scan, bind, and cleave target DNA [5]. In 2012, scientists showed that these two RNA components could be joined into a single-guide RNA (sgRNA). The sgRNA directs the Cas9 complex to the appropriate DNA sequence [5]. This permits Cas9 to search the DNA for a protospacer-adjacent motif (PAM). Once the PAM sequence is identified and the DNA is unwound, the sgRNA is matched to the appropriate target sequence in the DNA to confirm correct base pairing. Following verification, both the HNH and RuvC nuclease domains are activated to cleave the DNA, resulting in a double-strand break (DSB) [4, 5]. After the generation of a DSB, the cell has two major repair pathways available. One possible route is non-homologous end joining (NHEJ). Because it re-ligates damaged DNA ends without a repair template, NHEJ is a method well suited for gene knockdown. This process typically results in small insertions or deletions (indels) that may disrupt the reading frame. The other route is homology-directed repair (HDR), which is largely limited to the S and G2 phases of the cell cycle and hence functions with much lower efficiency than NHEJ, particularly in post-mitotic cells such as cardiomyocytes [4, 5]. The key steps involved in CRISPR-Cas9 gene editing are summarized in the illustration in Figure 1.

**Figure 1:**
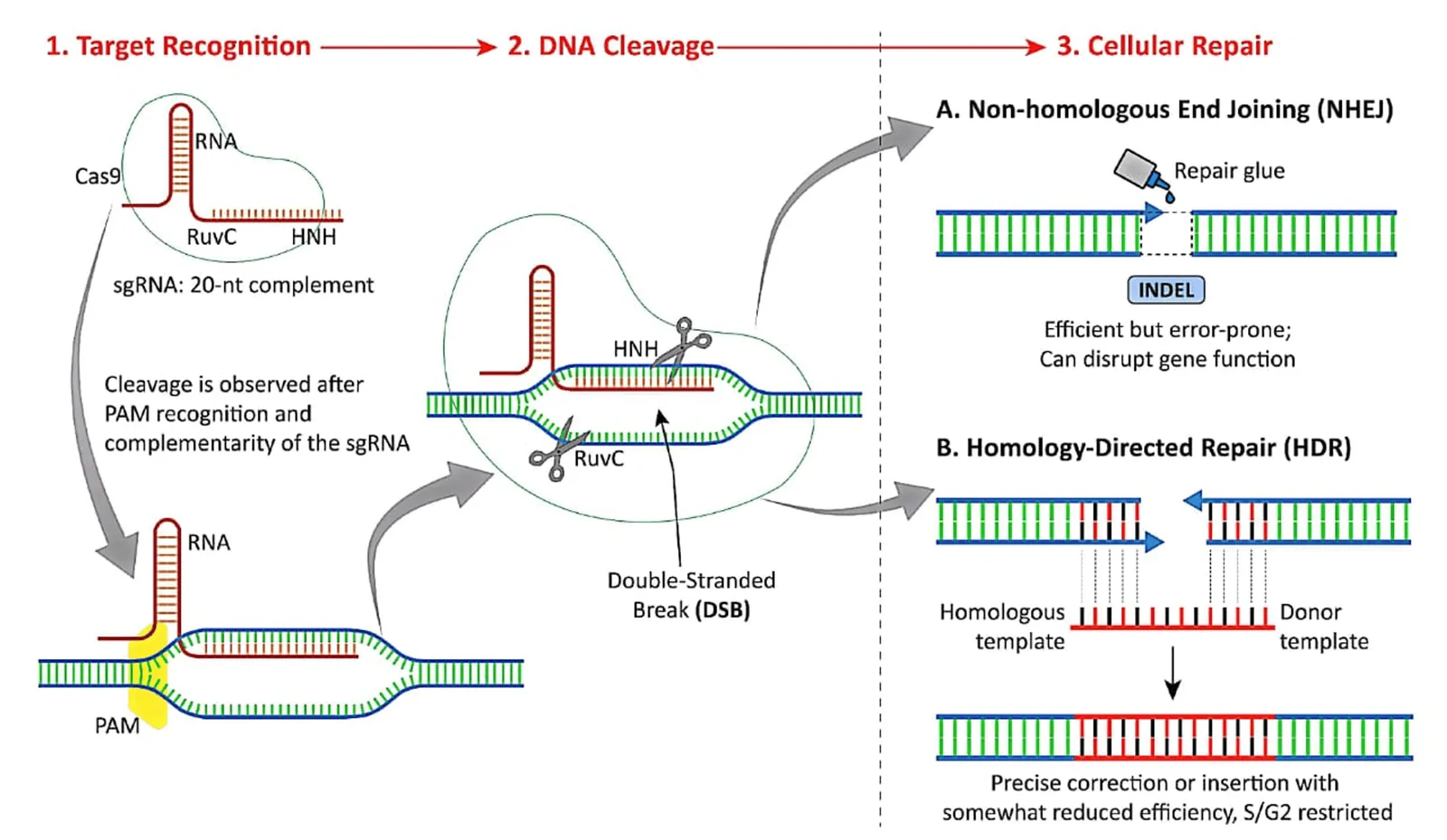
Key steps involved in CRISPR-Cas9 gene editing. Figure 1 shows the stepwise process of genome editing using CRISPR-Cas9. The first step is that the sgRNA enables the Cas9 enzyme to bind to the target DNA sequence adjacent to the PAM, allowing the system to locate the specific target. After binding, the HNH and RuvC domains of Cas9 cleave the DNA and produce a double-strand break (DSB). This break is then repaired by the cell through two main mechanisms: NHEJ and HDR. NHEJ is a rapid process but often leads to insertions or deletions (indels) that can disrupt gene function and allow gene knockdown. HDR can use a homologous donor template to precisely repair or insert a piece of genetic material. This pathway is less efficient, however, and is primarily active during the S and G₂ phases of the cell cycle. Together, these repair mechanisms constitute the molecular basis of CRISPR-Cas9-mediated therapeutic genome editing in precision medicine. This figure was created by the authors using Adobe Illustrator v25.4.1. Cas9: CRISPR-associated protein 9; RNA: Ribonucleic acid; RuvC: RuvC nuclease domain; HNH: HNH nuclease domain; PAM: Protospacer-adjacent motif; sgRNA: Single-guide RNA; DSB: Double-strand break; HDR: Homology-directed repair; NHEJ: Non-homologous end joining; S phase: DNA synthesis phase of the cell cycle; G₂ phase: Second gap phase of the cell cycle.

Initial clinical experience with classical gene therapy revealed significant limitations. It is well known that these techniques depended on the semi-random integration of viral vectors into the genome and carried a high risk of insertional mutagenesis, activation of oncogenes, and severe immunological responses [14]. In contrast, CRISPR-Cas9 permits far more targeted editing of the genome at particular sites. Its simplicity and programmability are major advantages. Producing new proteins for each target is not necessary; instead, new sgRNAs can be designed, which is quicker and less expensive [4]. This versatility and accuracy have made CRISPR-Cas9 a very attractive platform for exploring therapies for a variety of hereditary cardiovascular disorders [5]. However, conventional CRISPR-Cas9 genome editing, although revolutionary in its potential, still has several basic limitations that restrict its application as a therapy. One of these is that double-strand DNA break (DSB) induction can result in outcomes such as off-target insertions or deletions (indels), large genomic deletions or rearrangements, and activation of the DNA damage response, which have the potential to impair genomic integrity and safety [15]. In addition, the attractive precision of HDR-driven sequence repair is limited by the requirement for the S and G₂ phases of the cell cycle, greatly limiting editing efficiency in slowly dividing and post-mitotic cells such as cardiomyocytes [16]. Such constraints have motivated the development of next-generation CRISPR-based genome-editing approaches that are able to generate precise genetic modifications without the need for DSBs or donor DNA templates. Base editing and prime editing offer the potential to correct disease-causing mutations with higher precision and fewer DSB-associated risks than conventional CRISPR-Cas9-mediated genome editing [17]. A schematic illustrating the evolution of CRISPR-Cas9 to next-generation editing platforms is depicted in Figure 2.

**Figure 2:**
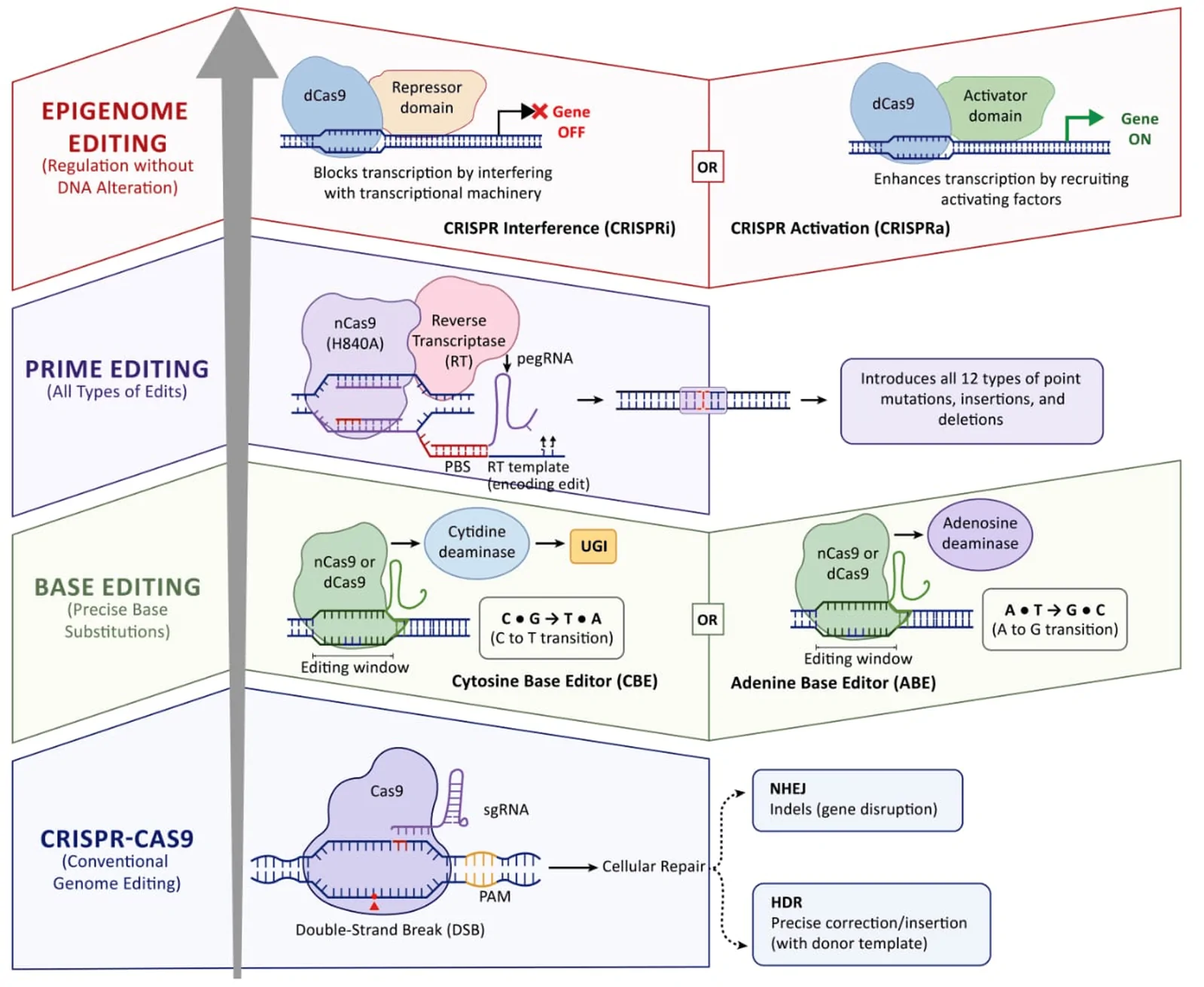
Overview of the evolution of CRISPR-based genome editing technologies. Conventional CRISPR-Cas9 causes double-strand DNA breaks that are repaired by NHEJ or HDR. Base editing enables precise single-base conversions without generating double-strand breaks and includes CBEs and ABEs. Prime editing extends the scope of editing to all types of point mutations, as well as small insertions and deletions, by combining a Cas9 nickase with a reverse transcriptase and a pegRNA. CRISPR-based transcriptional and epigenome editing utilizes catalytically inactive Cas9 (dCas9) linked to transcriptional repressors, activators, or epigenetic modifiers to control gene expression, enabling CRISPRi or CRISPRa without changing the underlying DNA sequence. This figure was created by the authors using Adobe Illustrator v25.4.1. ABE: Adenine base editor; CBE: Cytosine base editor; CRISPR: Clustered regularly interspaced short palindromic repeats; CRISPRa: CRISPR activation; CRISPRi: CRISPR interference; Cas9: CRISPR-associated protein 9; dCas9: Catalytically dead Cas9; HDR: Homology-directed repair; NHEJ: Non-homologous end joining; nCas9: Cas9 nickase; PAM: Protospacer-adjacent motif; PBS: Primer-binding site; pegRNA: Prime-editing guide RNA; RT: Reverse transcriptase; sgRNA: Single-guide RNA; UGI: Uracil glycosylase inhibitor.

Overview of emerging CRISPR-based genome editing

Base Editing

Base editing is a particular genome-editing technology that has been developed over time. It generally allows four of the twelve possible nucleotide substitutions at targeted loci, primarily through specific transition mutations, without generating DSBs [18, 19]. Base editors can either be conventional editors, including cytosine base editors (CBEs) and adenine base editors (ABEs), or expanded base editors, which enable additional types of nucleotide substitutions, including transversions [20, 21]. The base editor complex consists of a defective Cas9 protein (dCas9 or nCas9) and a nucleotide deaminase enzyme. A sgRNA directs the base editor complex to the target DNA sequence, and the nucleotide deaminase enzyme deaminates a specific nucleotide within the editing window [22]. CBEs catalyze C•G-to-T•A transitions by coupling a cytidine deaminase enzyme to a Cas9 nickase or dead Cas9. This complex deaminates cytosine in the target DNA to uracil [18, 22]. ABEs fuse an adenosine deaminase to a Cas9 nickase or dCas9 to catalyze A•T-to-G•C changes. The deaminase converts adenine to inosine and hence causes an A-to-G substitution. Base editors contain additional domains, such as uracil glycosylase inhibitors (UGIs) in CBEs, to limit undesired repair consequences and enhance editing purity [23]. These CRISPR-based technologies provide precise editing without DSBs, while related CRISPR-based regulatory approaches can provide reversible, sequence-specific transcriptional regulation without permanent changes in the DNA sequence [18]. They can target multiple locations simultaneously using a number of guide RNAs. They can be more efficient for specific nucleotide conversions and can be implemented in a wide range of cell types compared with conventional CRISPR-Cas9 [22]. Using these genome-editing techniques, human pluripotent stem cell-derived cardiomyocytes can be generated carrying specific genetic abnormalities to explore disease pathways in detail, e.g., in titin-linked cardiomyopathy or Barth syndrome [24]. *Prime Editing* Prime editing, a programmable editing approach, is a relatively recent genome-editing technology with improved precision and versatility. It may induce all 12 types of point mutations, as well as targeted small insertions and deletions at various loci, without producing DSBs or requiring an exogenous donor DNA template [25, 26]. Prime editing allows both indel mutations and all types of single-nucleotide changes [22]. Prime editors are directed by RNAs known as pegRNAs, which both specify the target site and encode the intended edit. This permits the reverse transcriptase domain to generate a modified DNA strand based on an RNA template [18]. Prime editors are not yet widely applied in cardiovascular research but are promising for the characterization of distinct genetic variations that cannot be characterized using conventional base editors [27]. *Epigenome Editing* Gene expression can be controlled by epigenetic changes. These processes include DNA methylation, which can silence gene expression, as well as histone acetylation and methylation. This can be achieved using a dCas9 fusion protein [18]. CRISPR interference employs a dCas9 fusion protein that binds to a sequence within a gene promoter or transcriptional enhancer, interfering with factors that normally bind that sequence to alter gene expression [28]. Another approach is CRISPR activation, which can be achieved by combining dCas9 with domains that can enhance gene expression or by modifying the guide RNA scaffold [24]. Moreover, CRISPRa and CRISPRi can also be utilized with guide RNAs to study various cell types [29]. Table 1 shows the comparative characteristics of current CRISPR-based genome-editing platforms.

**Table 1:** Comparative characteristics of current CRISPR-based genome-editing platforms. BE: Base editing; CRISPR: Clustered regularly interspaced short palindromic repeats; CRISPRa: CRISPR activation; CRISPRi: CRISPR interference; DSB: Double-strand break; dCas9: Catalytically inactive Cas9; HDR: Homology-directed repair; NHEJ: Non-homologous end joining; nCas9: Cas9 nickase; pegRNA: Prime-editing guide RNA; PE: Prime editing; sgRNA: Single-guide RNA.

Genome editing platform	Mechanism of Action	Editing capacity	Key Advantage	Major Limitation	Cardiovascular application	Author/References
CRISPR-Cas9	Cas9 nuclease is directed by a sgRNA to generate a site-specific DNA DSB, which is repaired by NHEJ or HDR.	Gene knockout, targeted insertions, deletions, and sequence correction through HDR	Simple design, highly programmable, efficient gene disruption, broad applicability, multiplex editing.	DSB-associated toxicity, off-target mutations, chromosomal rearrangements, insertion/deletion (indel) formation, and poor HDR efficiency in post-mitotic cells such as cardiomyocytes	Functional genomic studies, correction of inherited cardiovascular disease mutations (e.g., LMNA, TTN, PCSK9), disease modeling, and therapeutic gene disruption.	Bonowicz K et al. (2025) [5]
Base Editing	Cas9 nickase (nCas9) or catalytically inactive Cas9 (dCas9) is fused to a cytidine or adenine deaminase to directly convert one nucleotide into another without inducing DSBs.	Precise single-base substitutions (C→T and A→G; expanded editors now support additional base conversions)	High precision, minimal indel formation, no donor DNA required, suitable for correcting pathogenic point mutations, higher efficiency in non-dividing cells	Restricted editing window, bystander editing, PAM dependence, inability to generate large insertions or deletions	Correction of pathogenic variants responsible for inherited cardiomyopathies, channelopathies, familial hypercholesterolemia and generation of engineered cardiomyocyte disease models	Nishiga M et al. (2022) [18]
Prime Editing	Cas9 nickase is fused to reverse transcriptase and directed by a prime-editing guide RNA (pegRNA), which specifies both the target site and the desired edit without requiring DSBs or donor DNA.	All 12 base substitutions, precise small insertions, deletions, and combinations.	Broad editing versatility, high precision, reduced indel formation, no donor template required, capable of correcting mutations inaccessible to base editors	Lower editing efficiency than base editing in some settings, complex pegRNA design, delivery challenges, and limited in vivo experience.	Correction of diverse pathogenic cardiovascular mutations, functional validation of disease variants, and precision modeling of inherited cardiovascular diseases.	Anzalone AV et al. (2020) [23]
Epigenome editing (CRISPRi/CRISPRa)	Catalytically inactive dCas9 is fused to transcriptional activators, repressors, or epigenetic modifiers to regulate gene expression without changing the DNA sequence.	Reversible activation (CRISPRa) or repression (CRISPRi) of endogenous gene expression.	No permanent genomic modification, reversible regulation, reduced genotoxicity, suitable for functional genomics and therapeutic gene modulation	Does not permanently correct disease-causing mutations; variable efficiency depending on chromatin accessibility, repeated delivery may be required.	Investigation of cardiovascular gene regulatory networks, functional screening, modulation of disease-associated genes, and target validation.	Konermann S et al. (2015) [29]

Genetic basis of hereditary cardiovascular disorders

Many ICVDs are monogenic illnesses, i.e., caused by mutations in a single gene [2]. Often, just one copy of such a mutant gene is sufficient to cause disease phenotypes with a substantial impact on cardiovascular structure, function, or electrophysiology [30]. Many hereditary CVDs are inherited in an autosomal dominant manner with variable penetrance and expressivity [30, 31]. They are clinically relevant given their early onset, familial clustering, and association with catastrophic consequences, including heart failure and sudden cardiac death [30, 31], although they represent a smaller share of the total burden of cardiovascular disease. Cardiomyopathies represent an important subset of CVDs [32, 33]. HCM is one of the most common monogenic cardiovascular diseases [4]. Mutations in genes encoding proteins of the cardiac sarcomere, particularly *MYH7* and *MYBPC3*, are the most common causes of HCM. The *MYH7 *gene encodes β-myosin heavy chain, while the *MYBPC3* gene produces cardiac myosin-binding protein C [32, 33]. Mutations lead to functional alterations in cardiac muscle cells, cardiac muscle cell enlargement, decreased diastolic function, and arrhythmias [32]. Mutations in *MYH7* and *MYBPC3* have substantial penetrance and are linked with poor prognosis [32, 33]. DCM is defined by cardiac hypertrophy with systolic dysfunction [34, 35]. Genetic heterogeneity is more marked in DCM than in HCM [34]. Cytoskeletal and nuclear envelope proteins are often involved in DCM pathogenesis, and *LMNA* is one of the major genes implicated in DCM genetics [33, 34]. The *LMNA* gene encodes lamin A/C and is important for cardiomyocytes because it is involved in the regulation of cardiomyocyte mechanotransduction and gene expression [35]. Mutations in this gene can cause severe cardiomyopathy associated with heart failure, conduction abnormalities, and malignant arrhythmias [33, 35]. *TTN* mutations are responsible for up to 25% of DCM cases [36]. Cardiomyopathies have a very high degree of phenotypic overlap, and certain mutations in the *MYH7* gene may lead to HCM or DCM depending on the functional impact of the mutation [33, 34]. A further major category is that of hereditary arrhythmia disorders, sometimes called channelopathies [37]. Channelopathies are a family of hereditary diseases caused by mutations in genes encoding cardiac ion channels, resulting in electrical instability of the myocardium [38]. An archetypal example is long QT syndrome (LQTS), characterized by a prolonged QT interval and an increased propensity to develop life-threatening arrhythmias [38, 39]. Mutations in the cardiac sodium channel gene *SCN5A* are also involved in the pathophysiology of a number of hereditary arrhythmias, such as LQTS, Brugada syndrome, and cardiac conduction disorders, with evidence of clinical overlap among these disorders [38, 39]. Another prominent category is FH. This disorder is associated with abnormalities in lipid metabolism [12]. FH is one of the most frequent monogenic conditions, with an estimated frequency of 1:250-1:300 in the general population [31]. It is characterized by high levels of low-density lipoprotein cholesterol (LDL-C) and predisposes affected individuals to early coronary artery disease [31]. The most commonly affected genes are *LDLR*, *APOB*, and *PCSK9*, all of which are critical in lipid metabolism [31]. The identification of genes such as *MYH7*, *MYBPC3*, *LMNA*, and *SCN5A* has dramatically improved our understanding of the molecular basis of ICVDs [33, 34]. Mutations in these genes have been repeatedly associated with cardiomyopathies and arrhythmias, and these genes have well-established roles in the pathogenesis of these disorders [33, 34, 38]. Importantly, these genes are promising targets for genome-editing strategies, given that their disease-causing variants are well characterized and directly contribute to disease [2]. Many of these disorders are monogenic, indicating that modifying a single disease-causing gene may be sufficient to prevent or ameliorate these diseases [2].

In this context, CRISPR genome editing has emerged as a powerful potential therapeutic approach for managing genetic abnormalities responsible for certain hereditary cardiovascular disorders [2]. Gene repair may, for example, be employed to address *MYBPC3* mutations that are linked with cardiomyopathy [33]. Conversely, gene disruption may be used to address dominant-negative mutations in *MYH7* by reducing the expression of disease-causing variants [33]. Moreover, gene editing of *SCN5A* mutations may be helpful for the treatment of hereditary arrhythmia disorders [38]. It might restore normal ion channel function [32]. Gene editing of *LMNA* mutations may potentially be beneficial in the management of progressive myocardial dysfunction with conduction abnormalities [34]. Therefore, the specificity of the disease-causing mutations targeted by the CRISPR gene-editing system makes it a potential therapeutic option for treating hereditary cardiovascular diseases [2]. The main genes related to hereditary cardiovascular disorders and the prospective CRISPR targets are noted in Table 2.

**Table 2:** Major inherited cardiovascular diseases, their causal genes, pathogenic mechanisms, potential CRISPR-based therapeutic strategies, and status of evidence. APOB: Apolipoprotein B; Cas9: CRISPR-associated protein 9; CPVT: Catecholaminergic polymorphic ventricular tachycardia; CRISPR: Clustered regularly interspaced short palindromic repeats; DCM: Dilated cardiomyopathy; FH: Familial hypercholesterolemia; HDR: Homology-directed repair; HCM: Hypertrophic cardiomyopathy; LDL: Low-density lipoprotein; LDL-C: Low-density lipoprotein cholesterol; LDLR: Low-density lipoprotein receptor; LMNA: Lamin A/C; MYBPC3: Myosin-binding protein C3; MYH7: Myosin heavy chain 7; Nav1.5: Voltage-gated sodium channel Nav1.5; PCSK9: Proprotein convertase subtilisin/kexin type 9; RYR2: Ryanodine receptor 2; SCN5A: Sodium voltage-gated channel alpha subunit 5.

Disease Category	Disease	Gene	Protein/Function	Pathogenic Mechanism	Potential CRISPR-based Strategy	Evidence Status	References
Lipid disorder	FH	LDLR	LDL receptor; responsible for hepatic uptake and clearance of LDL particles	Loss-of-function variants reduce clearance of LDL, resulting in elevated LDL-C.	CRISPR/Cas9 gene editing of pathogenic LDLR variants in hepatocytes	Preclinical therapeutic evidence	[40]
Lipid disorder	FH	APOB	Apolipoprotein B; structural component of LDL and ligand for LDL receptor binding	A pathogenic variant can impair LDL receptor binding, resulting in impaired LDL clearance.	CRISPR/Cas9-mediated gene disruption/inactivation	Preclinical proof-of-concept; investigational	[41]
Lipid disorder	FH	PCSK9	Regulates degradation of LDL receptors	Gain-of-function variants cause increased LDL receptor degradation, resulting in elevated LDL-C levels.	CRISPR/base editing-mediated PCSK9 inactivation	Clinical-stage investigational	[11, 42]
Cardiomyopathy	HCM	MYH7	β-myosin heavy chain involved in sarcomere contraction	Pathogenic variants alter sarcomere function, leading to abnormally increased heart muscle contraction.	Allele-specific adenine base editing to correct pathogenic variants	Preclinical therapeutic evidence	[43]
Cardiomyopathy	HCM	MYBPC3	Cardiac myosin-binding protein C; involved in regulation of sarcomere contraction	Loss-of-function variants lead to reduced functional protein levels.	CRISPR/Cas9-mediated homology-directed repair (HDR) correction of pathogenic variants	Preclinical therapeutic evidence	[44]
Cardiomyopathy	DCM	LMNA	Lamin A/C; nuclear envelope structural protein	Pathogenic variants cause nuclear dysfunction, myocardial disease, conduction abnormalities, and arrhythmias.	Mutation-specific CRISPR/Cas9 allele correction	Preclinical therapeutic evidence	[45]
Channelopathy	Long QT syndrome type 3/arrhythmias	SCN5A	Nav1.5; voltage-gated cardiac sodium channel	Gain-of-function variants associated with LQT3 alter cardiac sodium current and prolong repolarization.	Adenine base editing to correct pathogenic variants	Preclinical therapeutic evidence	[46]
Channelopathy	CPVT	RYR2	Ryanodine receptor 2; a major intracellular calcium-release channel in cardiac cells	Pathogenic variants cause abnormal intracellular calcium handling and ventricular arrhythmias.	Allele-specific CRISPR/Cas9 editing/correction	Preclinical therapeutic evidence	[47]

CRISPR in cardiac disease modelling

The advent of CRISPR genome editing, with its simplicity, efficiency, and unprecedented precision, has provided a means of modeling mutations in mice and human iPSCs. This capability is being widely used in the cardiovascular field, primarily to understand the mechanisms underlying diseases such as cardiomyopathy, congenital heart disease, arrhythmias, and disorders of lipid metabolism and, subsequently, to open the door to therapeutic genome editing [48, 49, 50]. This development complements the impressive work done on minimizing modifiable risk factors, for example, hypertension and smoking, through both pharmacological and non-pharmacological approaches [4]. Human iPSCs can represent the genetics of their patient of origin and, in principle, can be differentiated into any desired cell type. This makes them excellent candidates for investigating the pathogenicity of underlying mutations and their associated cellular dysfunction in genetic diseases, as is being done in cardiovascular research [49, 51]. For example, iPSC-based modeling of clinically discordant siblings with a strong family history of HCM has been used to identify and functionally assess the effects of genetic modifiers in cardiomyopathies. It has demonstrated phenotypic similarities and differences, including biochemical and structural characteristics [51]. Similarly, patients with congenital heart diseases consider iPSC-based modeling useful in functional validation of the pathogenicity of some *GATA4* variants with previously unknown significance by detecting changes in their gene expression and alterations in conventional molecular pathways during cardiac differentiation. This information can also help direct subsequent therapy [50]. The creation of cardiovascular disease models using animal tissue can also be limited for other conditions, such as Long QT Syndrome (LQT1), due to significant differences in potassium channels between animal and human cardiomyocytes. The iPSC-CM models are more accurate tools for determining the exact pathogenic mechanisms arising from a wide range of mutations. Furthermore, they are useful for demonstrating the pharmacological effects of selected drugs, thereby screening for their effectiveness in managing LQT1 induced by *KCNQ1* mutations [52]. In the field of pharmacology, drug-target identification and validation have similarly been significantly advanced by CRISPR screening, which leverages its ability to disrupt specific genes and observe the resulting phenotypic changes. This method helps provide functional genomic insights into underlying disease processes, which can then be translated into therapeutic options. Integrating multiple CRISPR screens from different studies is essential for the discovery of new therapeutic targets [53, 54]. Furthermore, the evolution of the CRISPR-Cas9 system shows notable potential to affect transcriptional activity through dCas9. This potential is currently being explored in understanding human biology but has yet to be fully translated into therapeutics [54].

Therapeutic applications and preclinical studies

CRISPR-based genome editing for the therapy of hereditary cardiovascular disorders is at the forefront of precision medicine [5, 55]. The translational pathway for using CRISPR-Cas9 gene editing in humans is depicted in Figure 3. The first step is the identification of affected families and patients by screening for disease-causing mutations [56]. This is followed by the design of mutation-specific guide RNAs (gRNAs) and related Cas9 editing components [57], which are delivered to cardiac tissues using vectors such as adeno-associated viruses (AAVs) or lipid nanoparticles (LNPs). Further evaluation focuses on the restoration of normal cardiomyocyte activity, enhanced cardiac function, and screening for possible off-target effects [55]. The gene-editing revolution is advancing from the first generation of CRISPR-Cas9 systems to next-generation base and prime editors that can precisely correct pathogenic mutations and may allow gene therapy to transform cardiovascular medicine by targeting the molecular origins of disease. This technical progress is supported by the recent expansion in single-cell genomics, which enables remarkable resolution of cardiac disease and identifies new cell-specific targets [12].

**Figure 3:**
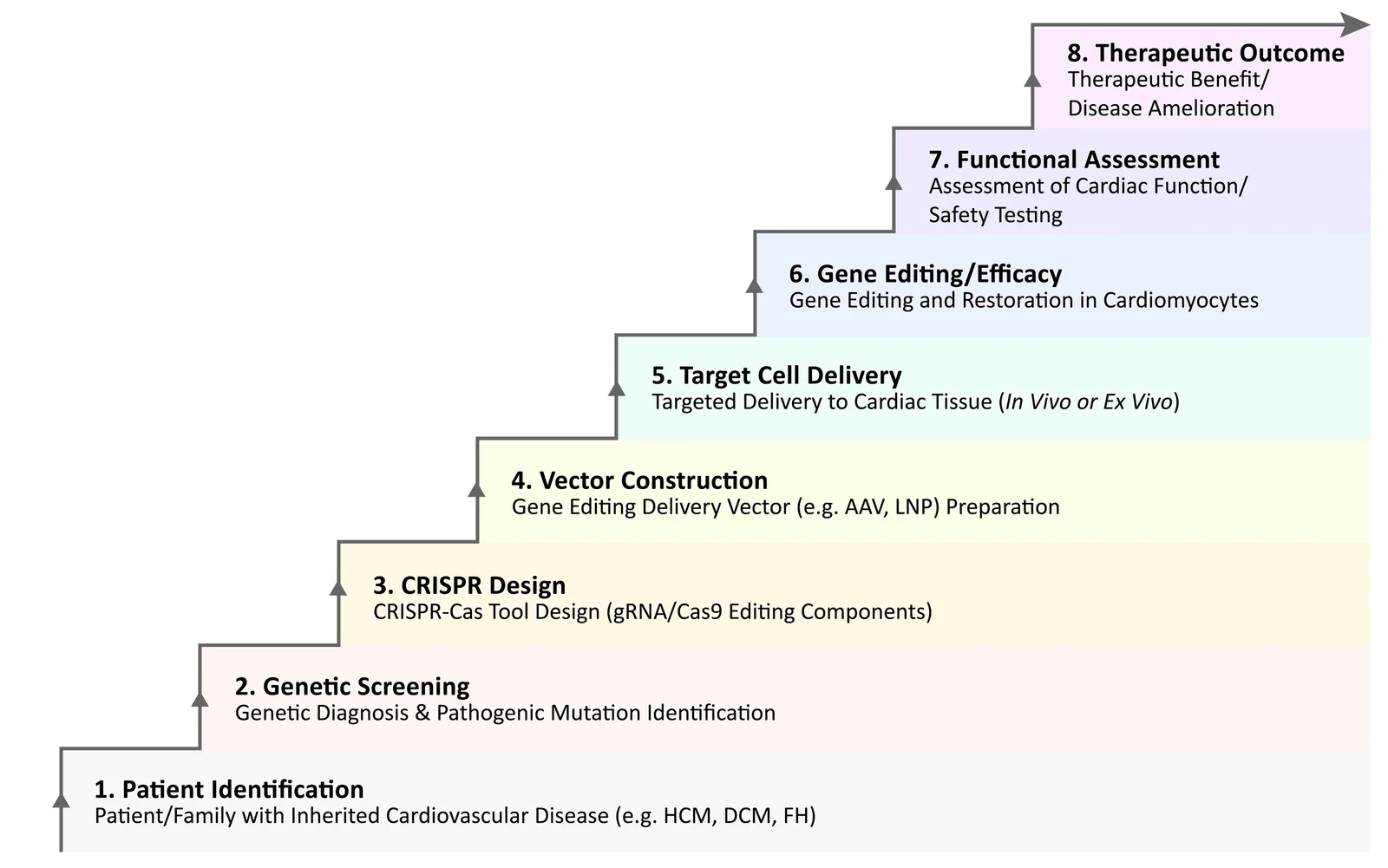
Stepwise workflow of CRISPR-Cas9-based therapeutic development for inherited cardiovascular diseases. This figure illustrates the workflow from patient identification to therapeutic outcomes in CRISPR-mediated cardiovascular gene editing. The process begins with the identification of patients or families with inherited cardiovascular diseases such as HCM, DCM, FH, and channelopathies. This is followed by genetic screening to identify pathogenic mutations. A CRISPR strategy specific to the target mutation is then designed. Delivery vectors such as AAVs or LNPs are subsequently developed to facilitate targeted delivery through in vivo or ex vivo approaches. The next steps are to evaluate gene-editing efficacy, assess cardiac function, and conduct safety testing. The intended outcomes are disease amelioration and clinical benefit. This figure was created by the authors using Adobe Illustrator v25.4.1. gRNA: Guide RNA; AAV: Adeno-associated virus; LNP: Lipid nanoparticle; HCM: Hypertrophic cardiomyopathy; DCM: Dilated cardiomyopathy; FH: Familial hypercholesterolemia.

HCM

The ability to create site-specific DSBs in DNA, followed by repair through two separate DNA repair pathways, NHEJ and HDR, permits different editing outcomes depending on the therapeutic goal. A notable example is a preclinical investigation in which sperm from healthy men heterozygous for a mutation in the *MYBPC3* gene, which causes HCM, were injected into egg cells from healthy females. sgRNA molecules were then put into freshly generated zygotes. gRNA was designed against the *MYBPC3* gene using CRISPR-Cas9 genome-editing technology. Many weeks later, the great majority of the embryos that resulted had lost the mutation-causing region of the *MYBPC3* gene without any other sections of the genome being affected [58]. Another important preclinical study used the adenine base editor (ABE8e) along with highly active Cas9, both supplied by AAV serotype 9 (AAV9) vectors [59]. Importantly, a single dose of dual-AAV9 vectors expressing ABE8e corrected >70% of cardiomyocytes in the ventricles of a heterozygous HCM mouse model carrying the R403Q mutation in the *Myh6* gene, and this treatment protected against deterioration of normal cardiac structure and function for an extended period [59]. Reichart D et al. found that in vivo repair of the R403Q mutation in HCM with a single therapy prevented disease development during long-term follow-up. In vivo adenine base editing or Cas9-mediated gene editing of the mutant allele may durably correct or inactivate a disease-causing variant in cardiomyocytes, preserving cardiac structure and function while reducing fibrosis and disease progression [59]. These findings provide robust support for the concept that genome editing may transition cardiovascular therapy from a symptom-centric approach to a potentially curative single treatment for hereditary disorders of the cardiovascular system.

DCM

Mutations in *TTN* are responsible for up to 25% of familial and 18% of sporadic non-ischemic DCM, which is a major cause of heart failure and an important indication for heart transplantation [36]. *TTN*-truncating variants (*TTN*tv) reduce the amount of normal titin protein (haploinsufficiency) and lead to the accumulation of shortened titin proteins within cells, compromising ubiquitin-dependent protein quality control [60]. Researchers have explored CRISPR-Cas9 therapy for *TTN*tv in a variety of preclinical models. A commonly used in vitro model is hiPSC-derived cardiomyocytes (hiPSC-CMs). Fomin A et al. evaluated titin expression in cardiac tissue from 113 patients with end-stage DCM. Both patient-derived cells with *TTN*tv mutations and cells with CRISPR-Cas9-generated *TTN*tv showed reduced levels of normal titin and contained truncated titin proteins compared with wild-type controls. In engineered cardiac muscle generated from hiPSC-CMs, CRISPR-Cas9 correction restored the decreased contractility caused by *TTN*tv, eliminated truncated titin proteins, and increased normal titin levels [60]. Moreover, scientists have generated hiPSC-derived cardiomyocyte models with I-band or A-band *TTN* mutations using CRISPR-Cas9 to study haploinsufficiency and dominant-negative effects. *TTN* mRNA levels were comparable in the two cell types relative to wild-type controls, but there were decreased quantities of full-length titin protein and detectable truncated protein products [61]. Grosch M et al. demonstrated an essential proof of concept for in vivo cardiac gene editing using transgenic and knock-in mouse models. In these models, AAVMYO and CRISPR-based editors were administered together to cardiac muscle in two knock-in mouse models to correct mutations in the cardiac splice factor *Rbm20*. This resulted in correction of more than 70% of cardiomyocytes. In young mice, it rescued the RBM20 localization deficiency in 75% of cells and improved the splicing of *RBM20* targets, including *TTN*. Three months post-therapy, cardiac size and function were restored, and there was no evidence of undesirable off-target editing [62]. Nishiyama T et al. generated *RBM20* (R636Q) knock-in mice to evaluate the therapeutic potential of adenine base editing (ABE) for *RBM20*-associated cardiomyopathy. Homozygous mutant mice recapitulated major features of DCM associated with human *RBM20* and developed severe cardiac dysfunction and early mortality. Systemic delivery of ABE components was performed using AAV9. The treated animals showed improved heart function on echocardiography, increased lifespan, and partial reversal of cardiac gene-expression patterns. These findings further corroborate the notion that in vivo base editing might ameliorate cardiac function in models of inherited cardiomyopathy and can correct deleterious splice-factor mutations [63]. Collectively, these preclinical studies demonstrate that CRISPR-Cas9 and other gene-editing technologies have the potential to correct pathogenic mechanisms related to *TTN*. These results are encouraging; however, the techniques are still in the preclinical stage, and several challenges, such as efficient and safe delivery in vivo, long-term durability of gene correction, immune responses, and minimizing off-target effects, among others, need to be addressed before the translation of CRISPR-based therapies for *TTN*-related DCM into clinical practice.

Mutations in *LMNA* are the second most prevalent genetic cause of DCM, representing approximately 5-6% of cases. They are associated with aggressive disease and can present early [64]. Researchers have employed CRISPR-Cas9 to develop iPSC-derived 3D cardiac organoids with a patient-specific *LMNA* mutation (c.937-1G>A) and generated corrected controls. These models demonstrated that CRISPR correction restored lamin A protein expression, cardiac differentiation, and calcium handling, with elevated systolic Ca²⁺ levels and faster calcium transients [65, 66]. A meta-analysis of 9 studies of 27 *LMNA* mutations in 154 iPSC lines concluded that CRISPR-corrected iPSC-derived cardiomyocytes had improved sarcomere structure, stronger contractions, normal calcium transient duration, lower APD90, and reversal of nuclear shape abnormalities compared with uncorrected *LMNA*-mutant controls [66]. Rather than direct mutation correction, an alternative method uses AAV-mediated gene therapy. NVC-001, an AAV serotype 9 gene therapy vector expressing a shortened version of the naturally occurring human SUN1 protein (dnSUN1), successfully targeted the LINC complex in iPSC-derived cardiomyocytes harboring an N195K *LMNA* mutation and rescued the arrhythmia phenotype. In an *Lmna* conditional knockout mouse model of *LMNA* DCM, treatment with NVC-001 resulted in 8-fold longer survival in treated mice (>300 days) versus untreated mice (<40 days), with stabilization of left ventricular function, rescue of conduction-system disease, and reduction in myocardial fibrosis [64]. Although further studies are needed to confirm long-term efficacy and safety, these findings establish the potential of gene-based approaches, including CRISPR-Cas9 correction, for *LMNA* cardiomyopathy.

FH

In animal models of FH, AAV-CRISPR/Cas9-mediated gene editing in vivo has been highly effective in correcting *LDLR* mutations and reducing atherosclerosis [40]. Homozygous LdlrE208X mice had severe atherosclerotic phenotypes on a high-fat diet. Treatment of hepatocytes with AAV-CRISPR/Cas9 targeting the nonsense mutation in the *LDLR* gene resulted in considerable restoration of LDLR protein expression [40]. Compared with control groups, the levels of serum total cholesterol, total triglycerides, and LDL cholesterol were significantly decreased in the AAV-CRISPR/Cas9 treatment group. Aortic atherosclerotic plaques were smaller and had less macrophage infiltration [40]. Another intriguing therapeutic target is *PCSK9*. People with loss-of-function mutations have lower plasma LDL levels and a lower risk of cardiovascular disease [67]. First-in-human clinical trials of base editing targeting *PCSK9* represent a milestone in the therapeutic translation of somatic genome editing [12]. In the phase 1 Heart-2 trial of VERVE-102, a single intravenous infusion of an in vivo ABE targeting *PCSK9* was tested in 35 participants with heterozygous FH or premature coronary artery disease across 6 dose cohorts. Dose-dependent reductions of up to 88% in *PCSK9* and 62% in LDL cholesterol were achieved, with no dose-limiting toxic effects, supporting further clinical development of one-time base-editing therapy for genetic hypercholesterolemia [11]. The angiopoietin-like protein 3 (*ANGPTL3*) may have a similar effect in homozygous and heterozygous FH patients independent of *LDLR* genotype and is another target for cholesterol reduction [68]. Administration of AAV for liver-specific in vivo base editing of *ANGPTL3* has been demonstrated to produce a near-complete reduction in circulating ANGPTL3, with a ~58% decrease in blood triglycerides and a ~61% reduction in total cholesterol at 4 weeks following AAV treatment [69]. Also under investigation are apolipoprotein C3 (*APOC3*), lipoprotein(a) (Lp(a)), and cholesteryl ester transfer protein (*CETP*), which together regulate multiple pathways in lipid metabolism [70].

Cardiac Channelopathies

Human iPSC-derived cardiomyocytes (iPSC-CMs) have become valuable tools for the study of channelopathies and the evaluation of CRISPR-based therapies [71]. These patient-specific cell models mimic the genetic background of patients and reproduce disease characteristics in vitro, such as prolonged action potentials, spontaneous arrhythmias, and other electrophysiological abnormalities reported in various channelopathies [71]. iPSC-CM models have been generated with *SCN5A* mutations associated with LQT3, and repair of these mutations by CRISPR/Cas9 has been shown to restore action potential duration and electrical properties toward normal [72]. CRISPR correction has also been shown to rescue the rapid delayed rectifier potassium current (IKr) in LQT2 models using *KCNH2*-mutated patient fibroblasts [73]. These findings demonstrate that ion channel function may be restored by genome editing [73]. With CRISPR/Cas9 technology, disease-causing mutations have been introduced into control iPSC-CMs, resulting in edited and unedited cell pairs [72]. This has improved the understanding of mutation-specific disease and medication responses in catecholaminergic polymorphic ventricular tachycardia (CPVT) and other channelopathies [74]. Studies using three-dimensional cardiac microtissues generated from genetically modified iPSC-CMs can mimic tissue-level electrical activity and arrhythmogenic pathways and may provide more physiologically relevant models [75]. CRISPR has also shown therapeutic potential in animal models of channelopathies. Pan X et al. showed that the *RYR2* R176Q mutation was targeted by CRISPR/Cas9 in live mice, completely preventing catecholamine-induced arrhythmias in heterozygous animals [47]. AAV9-mediated delivery of SaCas9 and mutation-specific gRNAs prevented arrhythmias in 100% of treated mice compared with 71% of untreated controls [47]. Deep sequencing showed that the editing was mutant-specific and did not generate detectable off-target alterations [47]. In cellular models of Brugada syndrome, correction of pathogenic variants associated with *SCN5A* dysfunction has been shown to restore electrophysiological abnormalities [76]. It is also significant that mutation-specific editing can target the mutant allele while preserving the normal allele [47]. Together, these data provide preclinical proof of concept that CRISPR-based genome editing can correct selected pathogenic variants and improve molecular, electrophysiological, and disease-associated phenotypes in cellular and animal models of inherited cardiac channelopathies. However, it remains to be seen whether these findings will translate into durable clinical benefit in patients, and further studies are required to establish long-term efficacy, safety, delivery, and clinical feasibility. The therapeutic applications of CRISPR-Cas9 and related genome-editing technologies in ICVDs are summarized in Table 3.

**Table 3:** Therapeutic applications of CRISPR-Cas9 and related genome-editing technologies in inherited cardiovascular diseases. AAV: Adeno-associated virus; AAV9: Adeno-associated virus serotype 9; ABE: Adenine base editor; ABE8e: Adenine base editor 8e; ANGPTL3: Angiopoietin-like protein 3; APOC3: Apolipoprotein C3; Cas9: CRISPR-associated protein 9; CPVT: Catecholaminergic polymorphic ventricular tachycardia; CRISPR: Clustered regularly interspaced short palindromic repeats; DCM: Dilated cardiomyopathy; FH: Familial hypercholesterolemia; HCM: Hypertrophic cardiomyopathy; HDR: Homology-directed repair; hiPSC-CMs: Human induced pluripotent stem cell-derived cardiomyocytes; IKr: Rapid delayed rectifier potassium current; iPSC: Induced pluripotent stem cell; KCNH2: Potassium voltage-gated channel subfamily H member 2; LDL-C: Low-density lipoprotein cholesterol; LDLR: Low-density lipoprotein receptor; LMNA: Lamin A/C; LV: Left ventricular; MYBPC3: Myosin-binding protein C3; MYH7: Myosin heavy chain 7; PCSK9: Proprotein convertase subtilisin/kexin type 9; RBM20: RNA-binding motif protein 20; RYR2: Ryanodine receptor 2; SCN5A: Sodium voltage-gated channel alpha subunit 5; TC: Total cholesterol; TG: Triglycerides; TTN: Titin.

Diseases	Target gene	Genome Editing Strategy	Experimental Model	Major Findings	Development Stage	Author/References
HCM	MYBPC3	CRISPR-Cas9 (mutation-specific editing)	Human embryos (zygotes)	Correction of the pathogenic MYBPC3 mutation, with a high proportion of embryos carrying the wild-type sequence	Preclinical	[58]
HCM	*MYH7* (R403Q)/Myh6	Adenine base editor (ABE8e) + AAV9	Heterozygous mouse model	More than 70% of cardiomyocytes were corrected, with prevention of hypertrophy and fibrosis and long-term preservation of cardiac function	Preclinical	[59]
DCM	TTN	CRISPR-Cas9	Patient-derived hiPSC-CMs	Titin expression was restored, truncated proteins were eliminated, and myocardial contractility was improved	Preclinical	[60]
DCM	*TTN* (I-band/A-band variants)	CRISPR-Cas9 disease modeling	hiPSC-CMs	Demonstrated haploinsufficiency and dominant-negative mechanisms in TTN-associated DCM	Preclinical	[61]
DCM	RBM20	Base editing (ABE) + AAVMYO	Knock-in mouse models	>70% editing efficiency, restored RBM20 localization, improved TTN splicing, and normalized cardiac function	Preclinical	[62]
DCM	*RBM20* (R636Q)	Adenine base editing	Knock-in mouse model	Improved ventricular function, corrected gene expression, and prolonged survival	Preclinical	[63]
DCM	LMNA	CRISPR-Cas9 correction	iPSC-derived 3D cardiac organoids	Restoration of lamin A expression, improved cardiac differentiation, and improved calcium handling	Preclinical	[65]
DCM	LMNA	CRISPR-corrected iPSC analysis	154 iPSC lines (meta-analysis)	Improved sarcomere organization, contractility, and calcium transients, with reversal of nuclear abnormalities	Preclinical evidence synthesis	[66]
DCM	LMNA	AAV9-mediated dnSUN1 gene therapy (NVC-001)	Mouse model	8-fold increase in survival, improved LV function, and reduced fibrosis	Preclinical	[64, 65]
FH/hypercholesterolemia	LDLR	AAV-CRISPR/Cas9	Mouse model	LDL receptor expression was restored, LDL cholesterol was reduced, and atherosclerosis was attenuated	Preclinical	[40]
FH/hypercholesterolemia	PCSK9	Base editing	Early human clinical studies	Durable reductions in PCSK9 and LDL-C following a single treatment	Clinical-stage investigational	[11, 67]
Lipid disorder	ANGPTL3	AAV-mediated base editing	Mouse model	Near-complete reduction of circulating ANGPTL3, with approximately 58% reduction in triglycerides and 61% reduction in total cholesterol	Preclinical	[68, 69]
Lipid disorders	*APOC3*, *LPA*, *CETP*	Emerging genome-editing targets	Experimental studies	Potential future therapeutic targets for lipid lowering	Early preclinical	[70]
Inherited cardiac channelopathies	SCN5A	CRISPR-Cas9 correction	hiPSC-CMs	Action potential duration and sodium channel function were restored	Preclinical	[71, 72]
Long QT syndrome type 2 (LQT2)	KCNH2	CRISPR-Cas9 correction	Patient-derived iPSC-CMs	Rapid delayed rectifier potassium current (IKr) and electrophysiological abnormalities were restored toward normal	Preclinical	[73]
CPVT	*RYR2* (*R176Q*)	CRISPR-Cas9 + AAV9	Mouse model	Eliminated catecholamine-induced ventricular arrhythmias with mutation-specific editing	Preclinical	[47]
SCN5A Brugada syndrome	SCN5A	CRISPR-Cas9	Animal model	Restoration of sodium channel function and normalization of cardiac repolarization	Preclinical	[76]
Multiple channelopathies	Multiple genes	CRISPR disease modeling	hiPSC-CMs and engineered cardiac tissues	Improved understanding of mutation-specific disease mechanisms and therapeutic responses	Preclinical	[71, 74]

Delivery Systems for Cardiovascular Genome Editing

Efficient transport of CRISPR components to target cells and tissues is a critical parameter for therapeutic effectiveness, since this directly affects editing efficiency and clinical translation. There are several important considerations that should inform the choice of a gene delivery vector, including the size of the genetic payload, target tissue, and duration of targeted gene editor expression [77]. Both viral and non-viral systems have been examined. AAV vectors are among the most commonly used delivery platforms for in vivo cardiovascular gene editing, in part owing to their high transduction efficiency and tropism for cardiac tissue. AAV-mediated delivery can lead to persistent expression of Cas9 and guide RNA, which is especially advantageous in post-mitotic cardiac tissue [77]. The AAV9 serotype can cross the vascular endothelium and is especially useful for genome editing of cardiomyocytes [78]. AAV8 is hepatotropic, which may be useful for investigations involving lipid metabolism and ASCVD [79]. However, AAV vectors are constrained by their small packaging capacity (~4.7 kb), which sometimes requires dual-vector delivery or the use of smaller Cas variants for larger constructs. The human immune response also presents additional challenges, primarily because of the high prevalence of pre-existing antibodies against commonly used AAV serotypes [80]. Non-viral delivery methods, including LNPs, electroporation, and virus-like particles, have various benefits, including transient expression of gene-editing components and potentially lower immunogenicity. LNPs show high efficiency in hepatocyte gene editing. LNPs can encapsulate mRNA or other genome-editing components and tend to target hepatocytes through interactions with serum proteins, making them useful for editing liver-expressed genes such as *PCSK9* and *ANGPTL3* [81].

Challenges and barriers to clinical translation

Off-Target Genome Editing Effects

Off-target effects have been classified into two main categories: one driven by similarity in genetic sequences and the other due to erratic Cas9 binding at unintended loci [82]. These off-target activities typically result from imperfect guide RNA-target pairing and bystander cleavage mediated by the Cas enzyme. The results of these off-target effects range from changes in chromosomal organization with consequent alteration of the function of normal physiology or gene signaling to predisposition to carcinogenesis [83]. Off-target effects differ across different editing systems and cell types. Genotoxicity studies reveal that the amount and effects of inadvertent editing are dependent on the cellular environment. Base editors enable targeted correction of point mutations without generating DNA DSBs but may also exhibit platform-specific genotoxicity [84]. Hyperactive CBEs display increased off-target activity and structural variant formation compared to conventional CRISPR-Cas9 in experimental models [84].

Delivery Efficiency to Cardiac Tissue

Currently, the efficient delivery of the CRISPR-Cas system to the cardiovascular system for safe and effective treatment of diseases remains, at best, a challenge, given its nature to alter genes and potentially change physiologic processes [48]. There are no established, readily available tools that have undergone clinical trials, and the majority are still experimental, currently being tested on animal models. Traditional methods such as the physical carrier methods are very limited in practical use. More recently developed tools being developed for delivering genetic alteration systems, such as AAV vehicles, nanoparticles, and virus-like particle tools, also encounter difficulties for delivery to cardiac tissues as well as other organ systems [85]. The use of nanoparticles for delivery of CRISPR systems is particularly plagued by the lack of cardiac surface markers that would enable the uptake of the nanoparticles [85]. Table 4 provides a summary of the principal CRISPR delivery platforms, their cargo, strengths, limitations, and respective cardiovascular applications.

**Table 4:** Delivery systems for cardiovascular genome editing. AAV: Adeno-associated virus; AAV8: Adeno-associated virus serotype 8; AAV9: Adeno-associated virus serotype 9; ASCVD: Atherosclerotic cardiovascular disease; Cas9: CRISPR-associated protein 9; CRISPR: Clustered regularly interspaced short palindromic repeats; DCM: Dilated cardiomyopathy; FH: Familial hypercholesterolemia; gRNA: Guide RNA; HCM: Hypertrophic cardiomyopathy; LNPs: Lipid nanoparticles; mRNA: Messenger RNA; PCSK9: Proprotein convertase subtilisin/kexin type 9; RNP: Ribonucleoprotein; sgRNA: Single-guide RNA; VLPs: Virus-like particles.

Delivery System	Cargo Delivered	Principal Advantages	Major Limitations	Representative Cardiovascular Applications	References
AAV9	Cas9, gRNA, base editors	Good cardiac tropism, effective cardiomyocyte transduction, and sustained expression	Host immune response and small packaging capacity (about 4.7 kb)	HCM, DCM, inherited arrhythmias	[77]
AAV8	Cas9, gRNA	Excellent hepatocyte tropism and effective liver gene editing	Small packaging capacity and immunogenicity limitations similar to those of AAV9	Familial hypercholesterolemia and lipid disorders	[79]
LNPs	mRNA, sgRNA, Cas9 RNPs	Lower immunogenicity, transient expression, and decreased risk of vector integration	Intrinsic cardiac targeting is limited	PCSK9, ANGPTL3; liver-directed editing	[81]
Electroporation	Cas9 RNPs, DNA plasmids	High editing efficiency ex vivo	Poorly suited for systemic in vivo delivery	Cell-based research and iPSC editing	[83]
VLPs	Cas9 RNP complexes	Transient delivery without the need for viral genome integration	Early-stage technology with limited clinical data	Emerging cardiovascular applications	[85]

However, base editor genes are larger than the packaging capacity of an AAV, their mRNAs are very lengthy and may create challenges for LNP-mediated transport, and recombinant production and purification of base editor proteins remain tough to do in high yield. This restriction has resulted in the creation of split-intein dual-AAV strategies that, however, bring their own set of challenges. [86] Prime editors are more than 6.3 kb in size, making in vivo distribution more complicated. Researchers have produced a compact prime editor lacking the RNase H domain, which achieved similar editing to full-length PE, although the size of prime editors limits the options for split sites inside Cas9, which directly influences editing efficiency. Similarly, for CBEs, even with split approaches based on trans-splicing intein strategies, the current split DNA cassettes are still above the AAV packing limit of 4.7 kb. [87]. The cellular production of AAV is a challenge that impacts both production efficiency and editing outcomes.

Immune Responses to CRISPR Components

CRISPR components have been shown in various tests to induce a number of innate and adaptive/humoral immune responses. The innate responses are thought to be initiated by guide RNA [88]. Some individuals may have also been pre-exposed to antigens contained within the CRISPR system, and these could potentially set off the adaptive immune system, causing aggravated reactions and destruction of the CRISPR system and eventual failure of the therapy [88]. It is estimated that more than 60% of the population has developed adaptive immunity against the components of CRISPR [4].

Ethical Concerns

Current ethical limitations involve the genetic alterations of somatic and germline genes. While somatic alterations really affect the individuals involved, germline alterations affect individuals as well as succeeding generations. The implications of negative genetic alterations could potentially have lasting impacts [89]. Within somatic editing, although the transference of altered genes is prevented, the process does not change the propensity for the reproduction of offspring with the defective genes, thereby extending the propagation of such conditions within the population [89]. Two commonly highlighted obstacles with germline alterations are the changes that are transferred to the next generation, which may become permanent, and the inability to obtain consent from succeeding generations [4, 89]. Although CRISPR technology is primarily being considered for the treatment and prevention of diseases, there are currently no limitations regarding its use for other purposes, and potentially wrongfully so, as different nations and people have differing opinions about the use of genetic modification tools [4].

Variants of Uncertain Significance (VUS) Interpretation

The issue of interpretation of genetic data from patients with ICVDs is still relevant. Currently, the clinical significance of about 40% of the variants in genes associated with ICVDs is unknown, indicating the need for new approaches to assessing their pathogenetic contribution [90]. This diagnostic uncertainty is an obstacle to designing patient-specific CRISPR therapeutics, because many identified variants may not be truly pathogenic.

Scalability and Manufacturing for Cardiac-Specific Delivery

A major bottleneck remains the production scale and total cost. The manufacturing pipelines and quality control processes for CRISPR delivery systems still need to be significantly improved to ensure consistency, safety, and reproducibility on a large scale [91]. The main limitation in practice is the capacity to generate sufficient material to deal with the global burden of inherited cardiomyopathies.

Future perspectives

The field of genome editing in cardiovascular medicine is promising, with opportunities for improved editing precision, delivery methods, and personalized medicine [6]. We anticipate that developments in next-generation platforms, such as high-fidelity Cas9 variants, base editors, and prime editors, will increase editing precision and reduce off-target effects and unwanted genomic modifications [7]. Such improvements might allow the correction of a broader range of harmful mutations linked with hereditary cardiovascular disorders, including cardiomyopathies, channelopathies, and FH, and efforts should be concentrated on the development of safer and more efficient delivery methods [8]. This optimization involves modified AAV vectors, LNPs, virus-like particles, and transient ribonucleoprotein delivery methods for enhanced cardiac-specific targeting with reduced immunogenicity and sustained nuclease expression [9]. Concurrently, artificial intelligence is being progressively used to improve guide RNA design, predict off-target effects, develop newer Cas proteins, and uncover new therapeutic targets using large-scale functional genomic analysis [92]. Additional developments in genome editing, along with advances in regulatory, manufacturing, and ethical oversight, would undoubtedly accelerate the translation of CRISPR from an experimental platform to clinical settings [93].

## Conclusions

The advent of CRISPR-Cas9 technology has ushered in a new chapter in cardiovascular precision medicine, allowing us to address the genetic root causes of inherited cardiovascular diseases rather than only treating their clinical features. Disease models, patient-derived induced pluripotent stem cell-derived cardiomyocytes, and animal studies increasingly demonstrate the significant potential of genome editing to repair harmful mutations related to cardiomyopathies, channelopathies, and FH. Furthermore, the introduction of next-generation editing platforms, such as base editing and prime editing, has substantially enhanced the accuracy, efficiency, and flexibility of genetic alterations while reducing the risks associated with double-strand DNA breaks. These are significant achievements, but hurdles remain before CRISPR-based therapies can become common therapeutic options for hereditary cardiovascular diseases. Barriers to clinical translation include off-target gene editing, concerns regarding effective delivery and tissue-specific targeting to the heart, immunogenicity, long-term safety, and ethical considerations. Future progress will require the integration of high-fidelity editing technologies, advanced delivery platforms, AI-assisted guide RNA design, and rigorous long-term safety evaluation. As these challenges are increasingly overcome, CRISPR-based therapeutics have the potential to transform the management of inherited cardiovascular diseases from predominantly symptomatic treatment toward personalized, mechanism-based interventions capable of modifying the fundamental genetic drivers of disease. Continued translational research will be important to establish whether this promise can be safely realized in clinical cardiovascular medicine.
